# A systematic review of the impact of sedation practice in the ICU on resource use, costs and patient safety

**DOI:** 10.1186/cc8956

**Published:** 2010-04-09

**Authors:** Daniel L Jackson, Clare W Proudfoot, Kimberley F Cann, Tim Walsh

**Affiliations:** 1 GE Healthcare, Pollards Wood, Nightingales Lane, Chalfont St. Giles, Bucks, HP8 4SP, UK; 2 Heron Evidence Development Ltd, Building 210A, Butterfield Technology and Business Park, Luton, LU2 8DL, UK; 3 Royal Infirmary of Edinburgh, Edinburgh, EH16 2SA, UK

## Abstract

**Introduction:**

Patients in intensive care units (ICUs) often receive sedation for prolonged periods. In order to better understand the impact of sub-optimal sedation practice on outcomes, we performed a systematic review, including observational studies and controlled trials which were conducted in sedated patients in the ICU and which compared the impact of changes in or different protocols for sedation management on economic and patient safety outcomes.

**Methods:**

We searched Medline, Embase and CINAHL online literature databases from 1988 to 15^th ^May 2008 and hand searched conferences. English-language studies set in the ICU, in sedated adult humans on mechanical ventilation, which reported the impact of sedation practice on cost and resource use and patient safety outcomes, were included. All abstracts were reviewed twice by two independent reviewers, with all conflicts resolved by a third reviewer, to check that they met the review inclusion criteria. Full-text papers of all included studies were retrieved and again reviewed twice against inclusion criteria. Data were doubly extracted from studies. Study aims, design, population, and outcomes including duration of mechanical ventilation, length of stay in ICU and hospital, costs and rates of mortality and adverse events were extracted. Due to heterogeneity between study designs and outcomes reported, no quantitative data synthesis such as meta-analysis was possible.

**Results:**

Included studies varied in design, patient population and aim, with the majority being before-after studies. Overall, studies showed that improvements in sedation practice, such as the introduction of guidelines and protocols, or daily interruption of sedation, were associated with improvements in outcomes including ICU and hospital length of stay, duration of mechanical ventilation, and costs. Mortality and the incidence of nosocomial infections were also reduced.

**Conclusions:**

Systematic interventions to improve sedation practice and maintain patients at an optimal sedation level in the ICU may improve patient outcomes and optimize resource usage.

## Introduction

The majority of mechanically ventilated patients within the ICU receive sedative drugs to decrease anxiety, ensure comfort and facilitate treatments. The optimal sedation level varies according to the medical condition of individual patients and their treatment needs. Typically, sedation level is measured by nurses or physicians according to simple scales, such as the Ramsay scale or Richmond agitation sedation scale (RASS), in order to titrate sedative drugs appropriately.

The extent to which sub-optimal sedation is a problem in practice within ICUs is unclear, both in terms of how frequently this occurs and its impact on outcomes. Optimum sedation is dependent on factors such as the type of patient, co-morbidities, illness severity and treatments required. Therefore, the clinical outcome of interest may influence the definition of optimum sedation. For example, optimum sedation for a short-term outcome, such as synchronisation with the ventilator, may not be identical to longer-term outcomes, such as ventilator-associated pneumonia, ICU length of stay or psychological sequelae of critical illness.

Sub-optimal sedation practice may affect both clinical and economic outcomes (such as patient length of stay). We sought to investigate the impact of sedation practice on both patient safety and economic outcomes through a systematic review of the publicly available literature. We included all relevant study designs (observational studies and controlled trials) that were conducted in sedated patients in the ICU and compared the impact of changes in or different protocols for sedation management on economic and patient safety outcomes.

## Materials and methods

### Searching

Medline, Embase and CINAHL databases were searched from January 1988 to 15 May 2008 using terms for sedation, ICU, sub-optimal sedation and sedation quality management. The standard Scottish Intercollegiate Guidance Network (SIGN) filters for randomised controlled trials (RCTs), economic studies and observational studies [1] were combined to capture all study designs relevant to the study question. The search strategy for Medline is provided as an example [see Additional file 1]. Conference proceedings from 2005 to 2008 were hand-searched for relevant studies. All results were uploaded into a bespoke internet SQL-based database.

### Selection criteria

Inclusion of studies was according to predetermined criteria. Studies had to have a population of adult patients, sedated and on mechanical ventilation within the ICU, and had to report the effect of sedation practice or quality on patient safety or economic outcomes. Studies that reported incidence rates of sub-optimal sedation were also included (data not shown). Short-term studies (only including patients sedated less than 24 hours) were excluded.

Only English-language studies were included. All abstracts were reviewed twice by independent reviewers, with all conflicts resolved by a third reviewer (either by agreement with one of the reviewers or by discussion in the case of disagreement between all three reviewers), to check that they met the review inclusion criteria. Full-text papers of all included studies were retrieved and reviewed against inclusion criteria. The data from included studies were then extracted, summarised and analysed.

### Data extraction

Data were extracted by two reviewers, with any differences reconciled by a third reviewer (either by agreement with one of the reviewers or by discussion in the case of disagreement between all three reviewers).

The following data were extracted if reported: country; sponsor; study design; patient population; study aim; study setting; number of patients in the study; details of comparisons made - such as between different treatment arms, or between different sedation monitoring systems; acute physiology and chronic health evaluation (APACHE) II or simplified acute physiology score (SAPS) II score; duration of mechanical ventilation; length of stay in the ICU; length of stay in hospital; time from end of sedation to extubation; duration of sedation; total/mean sedative drug dose; rate of unintended extubations; rate of re-intubations; rate of procedures to investigate reduced consciousness level; sedative costs; total costs (ICU and hospital); mortality rate; incidence of nosocomial pneumonia; and incidence of delirium.

### Quantitative data synthesis

Due to the wide range of included study types, no studies were suitable for quantitative data synthesis. This was anticipated in advance and confirmed on evaluation of the studies. This was due to heterogeneity in study design (before-after versus non-randomised comparative studies versus RCTs), outcome definition, intervention (type of sedation protocol or intervention applied) and patient population.

## Results

### Systematic review study flow

**Figure 1:**
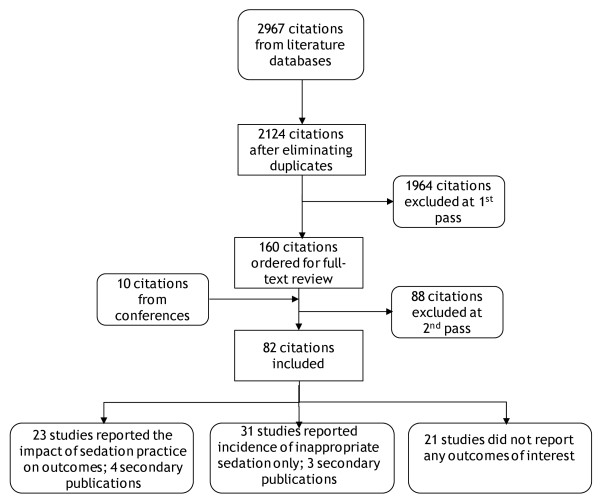
**The QUOROM diagram illustrates the flow of studies through the systematic review**. QUOROM, Quality of Reporting of Meta-Analyses.

The flow of studies through the systematic review is documented in the Quality of Reporting of Meta-Analyses (QUOROM) diagram in Figure 1.

Seventy five primary studies met the inclusion criteria, with seven secondary publications. Twenty one studies did not provide any data - either they did not contain data on the outcomes abstracted in this review or they did not provide this data in quantitative form. Twenty three studies reported data on the impact of sedation practice on outcomes and are reported in this review. The remainder reported the incidence of sub-optimal sedation and these data are reported elsewhere. Of the excluded studies, 8 were reviews, 10 were in a non-English language, not all patients were sedated in 10, 11 studies were not set in the ICU, patients were not mechanically ventilated in 21 and 28 studies did not report outcomes of interest.

### Summary of included studies

Nineteen studies investigated the effect of introducing a sedation management protocol or guideline on patient safety or economic outcomes. Of these studies, 15 were observational in design (typically before-after studies) and 4 were RCTs. Patient numbers ranged from 40 [2] to 1105 [3]. The predominance of relatively small, before-after studies highlights the limited quality of the evidence base.

The sedation protocols used varied, but generally included guidance on frequency and method of assessment of sedation adequacy, setting target sedation levels and/or choice of sedative drugs. Three studies specifically addressed the effect of sedation holds on outcomes [4-6].

In addition to the 19 protocol studies, an RCT of the bispectral index (BIS) monitor [7], and an RCT of intermittent lorazepam compared with continuous propofol [8] were included.

### Impact of sedation practice on duration of mechanical ventilation

Interventions to improve sedation practice were associated with a significant reduction in duration of mechanical ventilation in most studies. Sixteen studies examined the introduction of a sedation protocol on the duration of mechanical ventilation. Most studies used a before-after design [3,5,9-21], although one RCT of sedation holds was included [4]. All studies but one [17] observed a reduction in the average duration of mechanical ventilation in the protocol group, which was reported as statistically significant in eight studies [Table S1 in Additional file 1]. Absolute reductions in duration of ventilation varied due to differences between studies in average ventilation duration, presumably due to variations in case-mix. Reductions calculated as a percentage of control group value ranged from 10% [10] to 70% [5].

Two studies specifically investigated sedation holds: one RCT [22] and one observational study [5]. Sedation holds significantly reduced the duration of ventilation, by 1.5 times in the RCT, and by more than three times in the observational study. A second RCT of sedation holds reported the proportion of ventilator-free days over the 28-day study period was significantly higher in the sedation holds arm (11.6 versus 14.7) [6].

An additional RCT compared monitoring of sedation using the BIS monitor with standard sedation management [7]; this study reported that there was no difference in mean duration of mechanical ventilation between groups.

### Impact of sedation practice on weaning time

Four before-after studies [9,12,15,17,20] reported weaning time following protocol introduction (as a separate outcome from duration of mechanical ventilation). There was a significant reduction in weaning duration in two studies [12,20].

### Impact of sedation practice on length of stay in ICU

Improved sedation practice was associated with a reduced length of stay in the ICU. Sixteen studies reported the impact of a sedation protocol on length of stay in the ICU [Table S1 in Additional file 1]. There were two RCTs of sedation holds [4,6]; the remainder were observational studies (typically before-after design) [3,5,9-15,17-20,23]. All studies but one [17] found a protocol reduced length of stay; this was statistically significant in eight studies. Reductions as a percentage of control values varied from between 11% [11] to 64% [5]. Three studies specifically examined sedation holds and all found a significant reduction in length of ICU stay with sedation holds (of between 35% to 64%) [4-6].

Similar to ventilation duration, the RCT comparing the BIS monitor with standard sedation management [7] found no significant difference in median ICU stay between groups.

### Impact of sedation practice on length of stay in hospital

Improved sedation practice was associated with a reduction in hospital stay. Nine studies reported the impact of introducing a sedation protocol on length of patient stay in hospital [Table S1 in Additional file 1] [3-6,10,12,18-20]. All found a reduction in length of stay in the protocol group, which was significant in six studies [3,5,6,12,18,20]. Six studies examined the introduction of a general protocol for sedation improvement [3,10,12,18-20], while three studies compared a protocol of daily sedation holds with continuous sedation [4-6]; both types of comparisons found significant reductions in length of stay.

### Impact of sedation practice on sedation duration, sedative use and sedative costs

Six studies reported the impact of sedation protocols on the mean duration of sedation. Three indicated a reduction in duration of sedation with protocols: Chanques and colleagues [13] and De Jonghe and colleagues [14] reported a significant reduction after introduction of a protocol, while a third study reported a significant reduction in patients where guidelines were followed compared with those where guidelines were not followed [9], with reductions ranging from 39% [9] to 50% [14]. Three studies reported no change in duration of sedation with an intervention. These were an RCT of controlled versus empirical sedation, and two before-after studies of sedation protocols [2,17,20].

Five studies reported the impact of sedation protocols on sedative dosage. Quenot and colleagues [20] reported a significant decrease in daily drug dose for midazolam and propofol with protocol, and De Jonghe and colleagues [14] reported a similar finding for midazolam, but not fentanyl. An RCT reported a significant reduction in sedative drug dose with sedation holds in patients receiving midazolam [4] and two other studies found no significant change [10,18].

Seven studies reported the impact of sedation protocols on the costs of sedative agents used; all found a reduction in the costs of sedative agents with protocolised sedation, which was reported as significant in four studies [2,9,17,19], with values ranging from 22% to 94% of the cost for non-protocol managed sedation [Table S1 in Additional file 1].

### Impact of sedation practice on patient safety outcomes

#### Mortality

Thirteen studies reported the impact of sedation protocols on mortality [3-6,10-14,16,18-20], of which three specifically investigated sedation holds [4-6] (Table 1). Two studies of sedation holds were RCTs [4,6]; all remaining studies were before-after comparisons. Typically studies reported mortality rate for the period when patients were in the ICU, or in hospital, although Girard and colleagues reported 28-day and 1-year mortality rates [6]. Two sedation protocol studies showed a significant decrease in mortality following the introduction of a protocol [3,16], whereas the remainder showed no statistically significant change.

**Table 1:** Impact of sedation practice on patient safety outcomes

Study	Study type	Sample size	Comparison	Percentage mortality rate, timepoint for measurement and *P *value for comparison	Incidence of nosocomial pneumonia (%)	Accidental extubation rate (%)	Re-intubation rate
Marshall and colleagues 2008 [18]	Introduction of protocol (before-after study)	156	Before	Hospital mortality: 18%		10% (self-extubation)	11.5%
			
			After active pharmacist intervention	Hospital mortality: 15% *P *= 0.83		4% (self-extubation) *P *= 0.2	10.3% *P *= 0.8

Quenot and colleagues 2007 [20]	Introduction of protocol (before-after study)	423	Before	ICU mortality: 39% Hospital mortality: 45%	15% (ventilator -- associated pneumonia)	7 (unscheduled self-extubation)	
			
			After introduction of nurse-led sedation protocol	ICU mortality: 32% *P *= 0.19 Hospital mortality: 38% *P *= 0.22	6% (ventilator -- associated pneumonia) *P *= 0.005	11 (unscheduled self-extubation) *P *= 0.09	

Arabi and colleagues 2007 [10]	Introduction of protocol (before-after study)	207	Before education -- no protocol	ICU mortality: 20% Hospital mortality: 24%	28%		
			
			Before education -- protocol	ICU mortality: 18% Hospital mortality: 24%	29%		
			
			After education -- no protocol	ICU mortality: 23% Hospital mortality: 36%	11%		
			
			After education - protocol	ICU mortality: 13% *P *= 0.64 for all comparisons Hospital mortality: 23% *P *= 0.35 for all comparisons	11% *P *= 0.02		

Chanques and colleagues 2006 [13]	Introduction of protocol (before-after study)	230	Before	ICU mortality: 12%	14%	4% (self-extubation)	
			
			After monitoring of agitation	ICU mortality: 15% *P *= 0.76	9% *P *= 0.31; *P *= 0.03 for events/1000 ventilation days	2% (self-extubation) *P *= 0.65	

Burns and colleagues 2003 [3]	Introduction of protocol (before-after study)	1105	Before	38% (timepoint unclear)		10%	
			
			After introduction of outcomes management protocol	31% *P *= 0.02 (timepoint unclear)		7%	

Mascia and colleagues 2000 [19]	Introduction of protocol (before-after study)	156	Before	16.7% (timepoint unclear)			
			
			After introduction of protocol	17.6% *P *= 0.89 (timepoint unclear)			

Jakob and colleagues 2007 [16]	Introduction of protocol (before-after study)	300	After implementation of intervention 1 (change in ICU organisation)	ICU mortality: 19%			
			
			After implementation of intervention 2 (introduction of protocols for weaning)	ICU mortality: 8%			
			
			After implementation of intervention 1 (change in ICU organisation)	ICU mortality: 7% *P *= 0.02			

De Jonghe and colleagues 2005 [14]	Introduction of protocol (before-after study)	102	Before		8%		
			
			After introduction of protocol		20.4% *P *= 0.1		

Brattebo and colleagues 2002; 2004 [11,28]	Introduction of protocol (before-after study)	285	Before	ICU mortality: 27%		0	
			
			After introduction of protocol	ICU mortality: 22% (not significant)		0	

Brook and colleagues 1999 [12]	Introduction of protocol (before-after study)	321	Before	Hospital mortality: 36%			9%
			
			After introduction of protocol	Hospital mortality: 30%			13% *P *= 0.213

Kress and colleagues 2000; 2001; Schweickert and colleagues 2004 [4,22,29]	RCT of daily interruption of sedation	128	Continuous sedation	Hospital mortality: 47%	8% (ventilator-associated pneumonia)		
			
			Sedation interrupted daily	Hospital mortality: 36% *P *= 0.25	3% (ventilator-associated pneumonia)		

Girard and colleagues 2008 [6]	RCT of spontaneous breathing trials with and without daily interruption of sedation	335	Continuous sedation + spontaneous breathing trial	28-day mortality: 35% 1-year mortality: 58%		10% self-extubations 3% required re-intubation	14%
			
			Sedation interrupted daily + spontaneous breathing trial	28-day mortality: 28% *P *= 0.21 1-year mortality: 44% *P *= 0.01		4% self-extubation *P *= 0.03 2% required re-intubation *P *= 0.47	13% *P *= 0.73

Kollef and colleagues 1998 [5]	Continuous sedation vs intermittent sedation	242	Continuous sedation	Hospital mortality: 34%			15%
			
			Sedation interrupted daily	Hospital mortality: 30% *P *= 0.58			5% *P *= 0.005

Carson and colleagues 2006 [8]	RCT of intermittent lorazepam vs. continuous propofol	132	Lorazepam	Hospital mortality: 38%		2% self-extubations	16% reintubations
			
			Propofol	Hospital mortality: 37%		5% self-extubations *P *= 0.62	12% reintubations *P *= 0.59

Pandharipande and colleagues 2007a, b; 2006 [24,30,31]	RCT of dexmedetomidine vs lorazepam (RASS individualised to each patient)	103	Dexmedetomidine	28-day mortality: 17%		8%	
			
			Lorazepam	28-day mortality: 27% *P *= 0.18		4% *P *= 0.41	

RASS, Richmond agitation sedation scale; RCT, randomised controlled trial.

One of the sedation hold RCTs showed no significant effect on 28-day mortality, but a significant reduction in mortality after one year in the group where spontaneous breathing trials (SBTs) were paired with daily interruption of sedation in comparison with the control group [6]. The authors offered no explanation for this apparent late survival benefit. The other RCT and the before-after study comparing standard care with daily sedation holds reported non-significant decreases in mortality with daily sedation holds (47% versus 36% [4] and 34% versus 30% [5]). None of these studies were powered to detect mortality differences *a priori*.

One further study described mortality in relation to the introduction of intermittent bolus sedation compared with continuous sedation (both with daily sedation holds) and found no effect on mortality [8].

#### ICU-acquired pneumonia

Five studies reported the impact of sedation protocols on nosocomial pneumonia [4,10,13,14,20] (Table 1). Four were before-after studies [10,13,14,20]. Two showed significant reductions in the percentage of patients developing pneumonia [10,20]; a third study showed a significant impact on events per 1000 ventilation days but not on the proportion of patients with pneumonia [13]. The remaining study showed a non-significant increase in absolute incidence [4]. One RCT of daily sedation holds showed a reduction in pneumonia from 8% to 3% in the intervention group, which was not statistically significant. None of the studies were powered to detect changes *a priori*.

#### Extubation

Nine studies reported rates of self-extubation or accidental extubation [3,6,8,11,13,18,20,24] (Table 1). The only study which showed a significant difference in rates was the RCT by Girard and colleagues, which showed an excess of accidental extubations in the group randomised to daily sedation breaks and spontaneous breathing trials [6]. However, there was no difference in re-intubations, suggesting that many patients were ready for extubation, but did so unintentionally.

#### Reintubations

Five studies reported rates of re-intubations [5,6,8,12,18]; the only significant reduction was reported by the sedation holds study by Kollef and colleagues [5].

#### Spontaneous breathing trials

Two studies specifically described responses to SBTs in relation to sedation practice. In an RCT comparing intermittent lorazepam plus daily sedation break with propofol plus daily sedation break, the rapid shallow breathing index was significantly higher in patients randomised to lorazepam, suggesting that sedative drug may influence performance during SBTs [8]. In the RCT by Girard and colleagues, patients in the group randomised to daily sedation hold plus SBT had significantly higher RASS scores at the time of their first successful SBT than those randomised to SBTs and standard sedation practice [6]. These data suggest that both choice of sedative drug and sedation regimen potentially influence performance during SBTs.

#### Delirium

Surprisingly few studies evaluated the impact of sedation protocols on incidence of delirium. In the RCT comparing SBTs alone with SBTs plus daily sedation hold there was no difference in the incidence of delirium [6]. An RCT comparing sedation with dexmedetomidine with lorazepam found a significant reduction in prevalence or duration of delirium or coma, but not delirium alone, with dexmedetomidine [24].

### Procedures to investigate decreased consciousness

Only one study - the Kress RCT of sedation holds - specifically reported the incidence of procedures to investigate reduced consciousness level, and reported a significant reduction in diagnostic tests to assess changes in mental status (6 computed tomography (CT) scans versus 13 CT scans, 2 magnetic resonance imaging scans, and 1 lumbar puncture, *P *= 0.02) [4]. A second RCT of sedation holds also reported that the incidence of decreased consciousness such as coma was reduced by sedation holds, with a lower average duration of coma observed with sedation holds (*P *= 0.002) [6].

## Discussion

Our systematic review identified studies comparing the impact of different sedation practices on patient safety and economic outcomes. The included studies varied in study design, patient population, intervention, setting and time of study, preventing quantitative synthesis. Differences in individual study definitions of outcomes was also a major limitation that affected the potential for drawing firm conclusions from the data; however, it should be noted that comparisons made within studies (for example before-after or between groups) are still valid due to the fact that outcome definition can be assumed to be relatively consistent within studies. Nevertheless, the data strongly supported an association between specific interventions designed to optimise sedation and reduce duration of ICU stay and ventilation times. There were also suggestions of reductions in ICU-acquired pneumonia and short-term mortality in many studies.

Overall study quality was poor. The majority of studies were of before-after design and only four RCTs were identified. In addition, most studies enrolled relatively small numbers of patients and were not powered *a priori *for important patient-centred outcomes. The RCTs were relatively small-scale, with fewer than 500 patients included.

Before-after studies are less reliable forms of evidence than concurrent or randomised comparisons. They are subject to bias as other factors may change over time, which can affect outcomes. This is particularly important in critical care studies, where facilities, staffing and patient case-mix frequently change over time. One approach to correct for potential confounding is to adjust statistically, but this was not done in most studies. There is also a high risk of reporting bias, because studies where a protocol was introduced but had no beneficial effects on outcomes are less likely to be submitted or accepted for publication.

Baseline sedation practice will influence the plausibility of an intervention to improve outcomes. There was variation in patient characteristics and in baseline outcome values between studies, suggesting variation in sedation practice. However, limited details on baseline sedation practice, such as details of staffing levels and training, were provided, making it difficult to assess the impact of baseline practice on results.

A further possibility for bias is that it is not possible and is impractical to blind staff in protocol studies. This could result in a control group improvement associated with the study (the Hawthorne effect), but also potentially an artificial decrease in sedation quality in the control group compared with standard care. These factors emphasise the importance of clearly defining and describing 'standard care' in these studies.

It is possible that relevant studies may be missed. This review was confined to English-language publications, and therefore is biased towards the US, Europe and UK in focus. We also limited conference searching, which could have missed some of the relevant grey literature.

Despite these limitations, there was a consistent finding in both RCTs and before-after studies that interventions designed to optimise sedation practice improved outcomes. Systematic interventions reduced the duration of mechanical ventilation, length of ICU stay and the length of hospital stay. Fewer studies reported outcomes of total sedation duration, sedative dose and sedative cost. However, the available evidence indicated that these outcomes were also improved by protocol-directed sedation. The consistent numerical trend towards improvements across all ventilation and patient stay outcomes in most studies suggest that a systematic management approach is clinically important.

The available data also support an association between improvements in sedation practice and improved patient safety. Protocol-directed sedation tended to reduce mortality; although this was only statistically significant in 2 of 13 studies, the numerical trend was for a decrease and the lack of significance could be due to underpowered studies. Given the association between excessive sedation and adverse outcomes that are linked to patient mortality, such as nosocomial infection and delirium, it is biologically plausible that improving sedation practice could improve mortality.

Other important patient-safety outcomes were also reported by some studies. The most relevant of these was incidence of nosocomial pneumonia, which is associated with prolongation of ICU stay, poorer patient outcomes and increased costs. It is also a key quality indicator [25], and is linked to reimbursement in some healthcare settings. Despite the fact that most studies were not powered to detect differences in pneumonia incidence, there was a consistent suggestion of reductions in incidence with sedation improvements.

Less information was available for other safety outcomes. Increased rates of accidental extubation were not reported in most studies, and in the RCT that did report an increase in the intervention group the data suggested that this may have benefitted patients because re-intubation rates were similar to the control group [6]. Few studies systematically reported delirium. Delirium has been associated with sedation use, especially benzodiazepines, and is also associated independently with adverse outcomes in ICU populations [26]. One RCT found similar rates of delirium between the control group and a daily sedation hold group, where sedative drugs used were similar in both arms [6]. In contrast, a comparison of dexmedetomidine with lorazepam suggested decreased rates of delirium and coma with use of dexmedetomidine [24]. Therefore, choice of sedative agent may remain important even after controlling for other aspects of sedation practice. A detailed description and comparison of different sedative agents was not the aim of this review. In addition to these above-mentioned outcomes, a number of other important outcomes outside the scope of this review may be impacted by sedation quality, such as pain, anxiety or occurrence of post-traumatic stress disorder.

There was significant variation between studies in the approach taken to improving sedation. The management of sedation is complex and it is not possible to conclude from this review which component of the protocols was most associated with improved patient outcomes. The most recently published RCTs, which were the highest quality studies [4,6], indicate a strong association between daily cessation of sedation and improved outcomes. This finding supports the routine inclusion of this procedure in standard care, as is recommended in most recent guidelines and quality improvement initiatives [27]. It is also of note that few studies to date have addressed the question of sedation management in severely ill cases in the ICU, in whom sedation protocols are more difficult to apply and further work in this area is undoubtedly warranted.

Several technologies have been developed to measure consciousness level. These include the BIS and Entropy monitors. Although extensive literature is available on the association between these parameters and clinical sedation state, we only found one trial meeting our inclusion criteria. This was a small RCT using BIS, which found no benefit [7]. Therefore, additional studies are required to evaluate the impact of such technologies on patient-centred and economic outcomes. The association between practice-based interventions and improvements in clinical outcomes apparent from this systematic review indicate a need for formal outcome-based evaluation of health technologies in this area.

## Conclusions

Management of sedation during critical illness has been a focus of increased interest, particularly over the past decade. The overall quality of studies in this area is low. Nevertheless, this review indicates a strong association between systematic approaches to improving sedation and reduced duration of mechanical ventilation and length of ICU stay. The highest quality evidence suggests inclusion of daily sedation breaks is beneficial. Available evidence does not suggest an increase in adverse events with interventions that generally decrease sedation dose and duration, and risk of ICU-acquired pneumonia is probably decreased by such strategies. The quality and nature of standard care, and patient case-mix, are likely to be important determinants of the impact of changes within individual ICUs. Sedation practice is likely to strongly influence health care costs and improving sedation practice may not only benefit patients but also reduce such costs.

## Key messages

• Management of sedation during critical illness has been a focus of increased interest, particularly over the past decade.

• There is a strong association between systematic approaches to improving sedation and reduced duration of mechanical ventilation and length of ICU stay.

• The highest quality evidence suggests inclusion of daily sedation breaks is beneficial.

• Sedation practice is likely to strongly influence health care costs and improving sedation practice may not only benefit patients but also reduce such costs.

## Abbreviations

APACHE: acute physiology and chronic health evaluation; BIS: Bispectral Index Monitor; CT: computed tomography; QUOROM: Quality of Reporting of Meta-Analyses; RASS: Richmond agitation sedation scale; RCT: randomised controlled trials; SBT: spontaneous breathing trial; SIGN: Scottish Intercollegiate Guidance Network. SAPS: simplified acute physiology score.

## Competing interests

CP and KC are employed by Heron Evidence Development, which was commissioned by GE Healthcare to undertake this research. DJ is employed by GE Healthcare. GE is actively involved in the critical care area.

## Authors' contributions

DJ conceived the study and helped with manuscript revisions. CP designed and performed searches, extracted data and wrote the manuscript draft. KC researched and wrote the treatment guidelines section and assisted with data extraction for the main systematic review. TW provided expert clinical input and worked on manuscript revisions.

## Authors' information

Timothy Walsh is a Professor of Anaesthetics and Critical Care at Edinburgh University. Daniel Jackson is Head of Health Economics, EMEA at GE Healthcare. Clare Proudfoot is a Consultant at Heron Evidence Development Ltd, a health outcomes research consultancy. Kimberley Cann is a Health Outcomes Analyst at Heron Evidence Development Ltd.

## Supplementary Material

Additional file 1 **Additional material - Search strategy and impact of sedation practice on resource use**. This file contains two tables showing (1) the Medline search strategy and (2) a table of data from studies showing the impact of sedation practice on resource use. Click here for file
