# Supplementary material for: A systematic review of the impact of sedation practice in the ICU on resource use, costs and patient safety
Source: Crit Care. 2010 Apr 9;14(2):R59. doi: 10.1186/cc8956 (PMC2887180; doi:10.1186/cc8956)
Supplement: Additional file 1 — Additional material - Search strategy and impact of sedation practice on resource use. This file contains two tables showing (1) the Medline search strategy and (2) a table of data from studies showing the impact of sedation practice on resource use. [file cc8956-S1.docx]

Table S1: Impact of sedation practice on resource use (where more than one publication was reported for a single study, both publications are cited)

| Study | Study type | Study setting (ICU type and country) | Sample size (N) | Comparison | Health state of patients as measured by Apache II score or similar (mean, SD) | ICU length of stay in days (mean, SD) | Hospital length of stay in days (mean, SD) | Duration of mechanical ventilation in days (mean, SD) | Costs of sedation | Target sedation level (scale) |
| --- | --- | --- | --- | --- | --- | --- | --- | --- | --- | --- |
| Marshall 2008 [18] | Introduction of protocol (before-after study) | Medical ICU, USA | 156 | Before | 24.4 (7.3) | 15.8 (13.5) | 22.4 (14.6) | 0.59 (0.6) | NR | NR |
|  |  |  |  | After active pharmacist intervention | 22.7 (6.1) | 9.9 (8.6) p = 0.0021 | 15.4 (11.4) p = 0.001 | 0.31 (0.31)  p < 0.001 | NR | NR |
| Quenot 2007 [20] | Introduction of protocol (before-after study) | Medical ICU, France | 423 | Before | SAPS II: 45 (median) | 11 (median) | 21 (median) | 8 (median)  Weaning time: 2.7 (median) | NR | NR |
|  |  |  |  | After introduction of nurse-led sedation protocol | SAPS II: 51 (median) | 5 (median) p = 0.004 | 17 (median) p = 0.003 | 4.2 (median) p = 0.001  Weaning time: 1.38 (median)-p=0.01 | NR | Individual to patient (mainly II – III on Cambridge Scale) |
| Arabi 2007 [10] | Introduction of protocol (before-after study) | Medical/surgical ICU, Saudi Arabia | 207 | Before education– no protocol | 21 (1) | 13 (2) | 50 (7) | 12 (2) | NR |  |
|  |  |  |  | Before education – protocol | 23 (1) | 13 (1) | 55 (8) | 11 (1) | NR |  |
|  |  |  |  | After education – no protocol | 23 (1) | 12 (1) | 41 (7) | 10 (1) | NR |  |
|  |  |  |  | After education – protocol | 20 (1) | 10 (1) p = 0.42 | 40 (6) p = 0.34 | 8 (1) p = 0.21 (overall comparisons) | NR | 1 – 3 (SAS) |
| Chanques 2006 [13] | Introduction of protocol (before-after study) | Medical-surgical ICU, France | 230 | Before | SAPS II: 32 | 8.5 (median) | NR | 5 (median) | NR |  |
|  |  |  |  | After monitoring of agitation | SAPS II: 31 | 7 (median) p= 0.38 | NR | 2.7 (median)  p = 0.01 | NR | NR |
| Burns 2003 [3] | Introduction of protocol (before-after study) | Coronary, medical, neuroscience, surgical trauma and thoracic cardiovascular ICUs, USA | 1105 | Before | NR | 15 (median) | 22 (median) | 10 (median) | Cost per patient (direct): $51393 |  |
|  |  |  |  | After introduction of outcomes management protocol |  | 12 (median)  p < 0.0008 | 20 (median)  p < 0.0001 | 9 (median)  p < 0.0001 | Cost per patient (direct): $48168 | NR |
| Mascia 2000 [19] | Introduction of protocol (before-after study) | Medical and surgical ICUs, USA | 156 | Before | 19.5 | 19.1 | 34.3 | 13.2 | Significant reduction in mean cost per day for narcotics, benzodiazepines, propofol, and NMJ blockers. Maximal drug cost per day reduced for all but propofol < 24 hours |  |
|  |  |  |  | After | 21.2 | 9.9 (p values not reported) | 23.3 | 7.0 |  | NR |
| Anon 1999 [9] | Introduction of protocol (observational study) | ICU, USA | 94 | Guidelines not followed |  | 23.8 (18) | NR | 22.3 (13.8)  Weaning time: 5.4 (6.2) | $1468 (857) per patient. |  |
|  |  |  |  | Guidelines followed |  | 18.1 (8.7) (p values not reported) | NR | 13.4 (7.8)  Weaning time: 10.4 (10.9) | $581 (365) per patient. P < 0.05. | Individual to each patient |
| Devlin 1997 [15] | Introduction of protocol (before-after study) | Medical-surgical ICU, Canada | 100 | Before | 16.1 (5.7) | 4.3 | NR | 2.5  Weaning time: 0.67 | $81.5 (211.7) per patient  $11.27 (median per patient cost) |  |
|  |  |  |  | After | 22.4 (7.3) | 3.75 (not significant) | NR | 2 (not significant)  Weaning time: 0.75 | $18.12 (40.84) per patient  $3.55 (median per patient) | NR |
| Jakob 2007 [16] | Introduction of protocol (before-after study) | Medical-surgical ICU, Switzerland | 300 | Before | SAPS II: 30 | NR | NR | 0.75 | Swiss francs (CHF) 939 per patient |  |
|  |  |  |  | After implementation of intervention 1(change in ICU organisation) | SAPS II: 27 | NR | NR | 1 | CHF 598 per patient |  |
|  |  |  |  | After implementation of intervention 2 (introduction of protocols for weaning) | SAPS II: 27 | NR | NR | 0.5  P > 0.05 | CHF 533 per patient | NR |
| De Jonghe 2005 [14] | Introduction of protocol (before-after study) | Medical ICU, France | 102 | Before | SAPS II: 50.6 (16.0) | 15.0 (median) | NR | 10.3 (median) | Mean daily midazolam dose (79.1 52.7 mg) |  |
|  |  |  |  | After | SAPS II: 47.9 (15.2) | 8.0 (median) p = 0.43 | NR | 4.4 (median) p = 0.014 | Medan daily midazolam dose (55.7, 45.7 mg) | NR |
| Brattebo 2004, 2002 [11,28] | Introduction of protocol (before-after study) | Mixed surgical intensive care unit, Norway | 285 | Before | NR | 9.3 | NR | 7.4 | NR |  |
|  |  |  |  | After | NR | 8.3 (not significant) | NR | 5.3 (not significant) | NR | Individual to each patient |
| MacLaren 2000 [17] | Introduction of protocol (before-after study) | Medical-surgical-neurological ICU, Canada | 158 | Before | 22.7 (9.0) | 13.0 (8.1) – patients sedated > 48 h | NR | 9.9 (6.4)  patients sedated > 48 h  Weaning time: 1.6 (2.3) | Can$ 7.69 (5.29) per hour |  |
|  |  |  |  | After | 22.7 (6.9) | 13.9 (10.1) | NR | 11.0 (8.0)  Weaning time: 2.6 (4.1) | Can$ 5.68 (4.27) per hour  p< 0.01 | 3 – 4 (SAS) |
| Brook 1999 [12] | Introduction of protocol (before-after study) | Medical ICU, USA | 321 | Before | 23.2 (9.1) | 7.5 (6.5) | 19.9 (24.2) | 5.2 (6.4) | NR |  |
|  |  |  |  | After | 23.1 (8.5) | 5.7 (5.9)  p = 0.013 | 14 (17.3)  p < 0.001 | 3.7 (5.6)  p = 0.003  Weaning time: risk ratio of successful weaning 1.37 (95% CI: 1.19 – 1.58) | NR | Individual to each patient (Ramsay scale) |
| Tierney 1996 [21] | Introduction of protocol (retrospective observational study) | Medical-surgical ICU, Canada | 90 | Midazolam | 21.2 (7.1) | NR | NR | 6.2 | NR |  |
|  |  |  |  | Lorazepam | 21.9 (7.8) | NR | NR | 6.6 p = 0.77 | NR | NR |
| Bair 2000; Bobek 2001 [23,29] | Introduction of protocol (observational study) | Medical ICU, USA | 100 | Partial/ no adherence to guidelines | NR | 9 (median) | NR | NR | NR |  |
|  |  |  |  | Total adherence to guidelines | NR | 6 (median) p = 0.045 | NR | NR | NR | NR |
| Costa 1994 [2] | Empirical versus controlled sedation (RCT) | ICU | 80 | Empirical sedation | NR | NR | NR | NR | 12600 (1300) pesetas |  |
|  |  |  |  | Controlled sedation | NR | NR | NR | NR | 7900 (700) pesetas. p<0.05. | NR |
| Carson 2006 [8] | Intermittent lorazepam versus continuous propofol sedation | Medical ICU, USA | 132 | Intermittent lorazepam | 22.9 (7.7) | 10.4 (median) | 20 (median) | 8.4 | NR |  |
|  |  |  |  | Continuous propofol | 20.7 (7.3) | 8.3 (median) p = 0.20 | 18 (median) p = 0.55 | 5.8  p = 0.04 | NR | 2 – 3 (Ramsay) |
| Kress 2000; Kress 2001; Schweickert 2004 [4,22,30] | RCT of sedation holds | Medical ICU, USA | 128 | Continuous sedation | 22 (median) | 9.9 (median)  p = 0.02 | 16.9 (median)  p = 0.19 | 7.3 (median)  p = 0.004 | NR |  |
|  |  |  |  | Sedation interrupted daily | 20 (median) | 6.4 (median) | 13.3 (median) | 4.9 (median) | NR | 3 -4 (Ramsay) |
| Girard 2008 [6] | RCT of sedation holds | ICUs, USA | 335 | Continuous sedation (+spontaneous breathing trials) | 26.5 | 12.9 (median)  p = 0.01 | 19.2 (median)  p = 0.04 | NR | NR |  |
|  |  |  |  | Sedation interrupted daily (+spontaneous breathing trials) | 26 | 9.1(median) | 14.9 (median) | NR | NR | NR |
| Kollef 1998 [5] | Observational study of sedation holds | Medical ICU, USA | 242 | Continuous sedation | 20.2 (6.5) | 13.5 (33.7) | 21.0 (25.1) | 7.7 (7.9) | NR |  |
|  |  |  |  | Sedation interrupted daily | 21.2 (8.9) | 4.8 (4.1) p < 0.001 | 12.8 (14.1) p < 0.001 | 2.3 (3.2) p < 0.001 | NR | 3 (Ramsay) |
| Weatherburn 2007 [7] | RCT of BIS vs standard care | Surgical and general ICU, Australia | 50 | BIS | 14 (median) | 12 (median) | NR | 7.0 (0.6) | NR |  |
|  |  |  |  | Standard care | 14 (median) | 8 (median)  p = 0.2 | NR | 7.0 (0.8) p = 0.71 | NR | NR |

ICU: Intensive care unit; SD: standard deviation; NR: not reported; SAS: Sedation agitation scale

Table S2: Search strategies in Medline

| # | Search History | Results |
| --- | --- | --- |
| 1 | Randomized controlled trials/ | 54768 |
| 2 | Randomized controlled trial.pt. | 257701 |
| 3 | Random allocation/ | 61477 |
| 4 | Double blind method/ | 98005 |
| 5 | Single blind method/ | 12139 |
| 6 | Clinical trial.pt. | 452040 |
| 7 | exp Clinical Trial/ | 548580 |
| 8 | or/1-7 | 642453 |
| 9 | (clinic$ adj trial$1).tw. | 127359 |
| 10 | ((singl$ or doubl$ or treb$ or tripl$) adj (blind$3 or mask$3)).tw. | 97998 |
| 11 | Placebos/ | 27373 |
| 12 | Placebo$.tw. | 115124 |
| 13 | Randomly allocated.tw. | 10802 |
| 14 | (allocated adj2 random).tw. | 641 |
| 15 | or/9-14 | 282010 |
| 16 | 8 or 15 | 752691 |
| 17 | Case report.tw. | 140604 |
| 18 | Letter.pt. | 647165 |
| 19 | Historical article.pt. | 251094 |
| 20 | Review of reported cases.pt. | 0 |
| 21 | Review, multicase.pt. | 0 |
| 22 | or/17-21 | 1030734 |
| 23 | 16 not 22 | 735157 |
| 24 | Economics/ | 25665 |
| 25 | "costs and cost analysis"/ | 36882 |
| 26 | Cost allocation/ | 1853 |
| 27 | Cost-benefit analysis/ | 43343 |
| 28 | Cost control/ | 17864 |
| 29 | Cost savings/ | 6003 |
| 30 | Cost of illness/ | 10553 |
| 31 | Cost sharing/ | 1390 |
| 32 | "deductibles and coinsurance"/ | 1185 |
| 33 | Medical savings accounts/ | 385 |
| 34 | Health care costs/ | 16778 |
| 35 | Direct service costs/ | 849 |
| 36 | Drug costs/ | 8558 |
| 37 | Employer health costs/ | 988 |
| 38 | Hospital costs/ | 5606 |
| 39 | Health expenditures/ | 10181 |
| 40 | Capital expenditures/ | 1834 |
| 41 | Value of life/ | 5036 |
| 42 | exp economics, hospital/ | 15495 |
| 43 | exp economics, medical/ | 11745 |
| 44 | Economics, nursing/ | 3838 |
| 45 | Economics, pharmaceutical/ | 1915 |
| 46 | exp "fees and charges"/ | 23838 |
| 47 | exp budgets/ | 9844 |
| 48 | (low adj cost).mp. | 11749 |
| 49 | (high adj cost).mp. | 5291 |
| 50 | (health?care adj cost$).mp. | 1925 |
| 51 | (fiscal or funding or financial or finance).tw. | 47316 |
| 52 | (cost adj estimate$).mp. | 914 |
| 53 | (cost adj variable).mp. | 25 |
| 54 | (unit adj cost$).mp. | 931 |
| 55 | (economic$ or pharmacoeconomic$ or price$ or pricing).tw. | 103793 |
| 56 | or/24-55 | 323929 |
| 57 | Epidemiologic studies/ | 4081 |
| 58 | exp case control studies/ | 392636 |
| 59 | exp cohort studies/ | 675888 |
| 60 | Case control.tw. | 44129 |
| 61 | (cohort adj (study or studies)).tw. | 38594 |
| 62 | Cohort analy$.tw. | 1912 |
| 63 | (Follow up adj (study or studies)).tw. | 28751 |
| 64 | (observational adj (study or studies)).tw. | 18579 |
| 65 | Longitudinal.tw. | 85617 |
| 66 | Retrospective.tw. | 157285 |
| 67 | Cross sectional.tw. | 84687 |
| 68 | Cross-sectional studies/ | 89096 |
| 69 | or/57-68 | 1205940 |
| 70 | anesthesia/ or anesthesia recovery period/ or conscious sedation/ or deep sedation/ | 42426 |
| 71 | "Hypnotics and Sedatives"/ | 17039 |
| 72 | (an?esthe$ or sedat$).tw. | 263302 |
| 73 | or/70-72 | 283696 |
| 74 | Intensive Care Units/ | 23061 |
| 75 | intensive care unit.tw. | 34347 |
| 76 | ICU.tw. | 16202 |
| 77 | Critical Care/ | 19510 |
| 78 | or/74-77 | 68838 |
| 79 | 23 or 56 or 69 | 2049662 |
| 80 | Respiration, Artificial/ | 29624 |
| 81 | mechanical ventilation.tw. | 16790 |
| 82 | (mechanical$ or artificial$).tw. | 223073 |
| 83 | (ventilat$ or respir$).tw. | 322408 |
| 84 | 82 and 83 | 34236 |
| 85 | Intubation, Intratracheal/ | 23057 |
| 86 | intubat$.tw. | 29353 |
| 87 | 80 or 81 or 84 or 85 or 86 | 87382 |
| 88 | 73 and 78 and 79 | 1979 |
| 91 | (over$ or under$ or inappropriat$ or incorrect$).tw. | 3228742 |
| 95 | "quality of health care"/ or guideline adherence/ | 50304 |
| 96 | (audit or guideline or algorithm or protocol or target or manag$ or outcome$).tw. | 1367494 |
| 97 | 95 or 96 or 91 | 4102156 |
| 98 | 88 and 97 | 1485 |
| 99 | limit 98 to yr="1988 - 2008" | 1447 |
